# miR-375 is cold exposure sensitive and drives thermogenesis in visceral adipose tissue derived stem cells

**DOI:** 10.1038/s41598-022-13610-6

**Published:** 2022-06-10

**Authors:** Claudine Seeliger, Tanja Krauss, Julius Honecker, Laura Aline Mengel, Lise Buekens, Alberto Mesas-Fernández, Thomas Skurk, Melina Claussnitzer, Hans Hauner

**Affiliations:** 1 School of Medicine, Institute of Nutritional Medicine, Technical University of Munich, Freising-Weihenstephan, Germany; 2 ZIEL Institute for Food and Health, Technical University of Munich, Freising-Weihenstephan, Germany; 3 Else Kröner-Fresenius Center for Nutritional Medicine, School of Life Sciences, Technical University of Munich, Gregor-Mendel-Str. 2, 85354 Freising, Germany; 4 Broad Institute of MIT and Harvard, Cambridge, MA 02142 USA; 5 Harvard Medical School, Harvard University, Boston, MA 02215 USA

**Keywords:** Cell biology, Molecular biology, Biomarkers, Health care

## Abstract

Activation of brown adipose tissue may increase energy expenditure by non-shivering thermogenesis. Cold exposure is one of the options to activate brown adipocytes. To link changes in energy metabolism with microRNA expression (miRNAs), we analyzed 158 miRNAs in serum of 169 healthy individuals before and after cold exposure. Validating the results of a miRNA array, a significant down-regulation of miR-375 after cold exposure (*P* < *0.0001*) was detected. These changes went along with a significant negative correlation between miR-375 and visceral adipose tissue (VAT) mass *(P* < *0.0001)*, implicating a specific function of miR-375 in this depot. Significantly higher expression levels of miR-375 were found in VAT in comparison to subcutaneous fat (SAT). Using in silico prediction, we identified putative miR-375 target genes involved in the thermogenesis pathway. Cold-stimulation of subcutaneous and visceral pre-adipocytes (PACs) led to significantly higher expression levels of FABP4, FGF21, PPARGC1A and PRDM16 in VC-PACs. Analyzing miR-375 knock down and cold stimulated VC-PACs revealed a significant up-regulation of thermogenesis associated genes PPARGC1A, ELOVL3 and PRDM16. In summary, our findings identified miR-375 as a potential adipogenic and thermogenesis-associated miRNA exclusively acting in visceral adipose tissue.

## Introduction

Adipose tissue (AT) is a remarkably dynamic organ with profound effects on whole body (patho)-physiology^1^. AT plays a major role in nutrient homeostasis, as it stores and releases energy dependent on availability and demand. Further, AT is an endocrine organ secreting serum factors like leptin, TNF-α, MIF and PAI-1, beyond others^2–4^. As the most common classification scheme AT throughout the human body is subdivided into subcutaneous (SAT) and visceral adipose tissue (VAT). While SAT far exceeds VAT mass, expansion of VAT is considered metabolically more harmful compared to its subcutaneous counterpart^1^. Besides different associations with metabolic disease, the two depots also differ in their developmental origin and cellular composition^5^. Adipocytes themselves can be divided into at least two subtypes: (1) White, unilocular lipid-storing adipocytes are the predominant type of fat cell in the human body and (2) brown, multilocular adipocytes are characterized by expressing uncoupling protein 1 (UCP1) and are able to dissipate energy in the form of heat upon cold exposure. Likewise, there is growing evidence that multiple types of thermogenic (UCP1 +) adipocytes exist. Brown adipocytes are found within distinct brown adipose tissue (BAT) depots. Active BAT was long thought to be present mainly in rodents and human infants, but it was recently shown to be functional also in healthy adults^6–9^. Mild cold exposure is known to activate non-shivering thermogenesis (CIT), thereby increasing energy expenditure in humans with residual BAT^10^. Due to its ability to oxidize glucose and lipids, BAT activation is reported to exert beneficial effects on glucose and lipid metabolism^11,12^. Recent studies suggest that activating BAT may increase energy expenditure by thermogenesis and, therefore, may affect total energy homeostasis^6,13^. BAT in humans is primarily found around the neck and in the supraclavicular region. Recent single cell sequencing studies suggest that beige/brown like progenitors are also present in typical white fat depots with a preference towards VAT^14,15^. In rodents, prolonged cold exposure or adrenergic signaling can provoke the appearance of clusters of UCP-1 + cells with a brown fat-like morphology within white fat depots. This suggests that beige cells are uniquely programmed to be bifunctional, suited for energy storage in the absence of thermogenic stimuli, but fully capable of turning on heat production when appropriate signals are received^16^. The induction of browning is considered a potential strategy to combat obesity and recent research has unraveled cellular and molecular mechanisms that are involved in the process of thermogenesis. Recent studies also suggest that miRNAs are associated with the regulation of thermogenesis. However, these studies were performed mainly in mice or cell culture and have not been confirmed in human intervention studies^17–20^.

miRNAs are non-coding, short RNA segments of approximately 22 nucleotides. They control mRNA expression post-transcriptionally by forming an RNA-induced silencing complex (RISC), that either leads to mRNA cleavage or mRNA degradation and/or translation repression^21,22^. The miR-182-miR-203 cluster as well as miR-196a-5p and miR-328 were previously identified as positive regulators of brown/beige adipocyte development^23–27^. In contrast, the miRNA cluster miR-106b-93 was identified as a negative regulator of brown adipocyte differentiation^28^. In addition, miR-125b affects mitochondrial biogenesis and impairs brite adipocyte formation and function^29^. To date, little is known about miRNA changes in serum during cold acclimatization and their impact at the cellular level. The group of *Chen *et al*.* showed a negative correlation of exosome derived miR-92a with BAT activity in mice and a small cohort of human individuals undergoing cold exposure (CE)^19^. This miRNA was also found to be elevated in obesity and to be decreased after bariatric surgery^30^. Recently, our group reported that adult humans show high variability in resting energy expenditure (REE) after a standardized mild cold exposure^31^. An additional study revealed an increase in the expression of the adipose browning marker gene *CIDEA* in SAT from female but not in male participants^32^.

To our knowledge, no detailed miRNA analysis in serum collected during a human study applying cold exposure has been conducted so far. The aim of our study was to investigate changes in serum miRNA patterns upon cold exposure using a qPCR-based screening approach.

After target validation in a large cohort of 169 individuals, we studied the role of miR-375 in browning and its effect on thermogenesis related genes using a cell culture based approach. We identified putative target genes of miR-375 in human adipocytes and our study confirms the importance of this miRNA as a potential target for the thermogenesis pathway.

## Results

### Identification of differentially expressed miRNAs in serum of individuals undergoing cold exposure using a miRNA array

**Figure 1:**
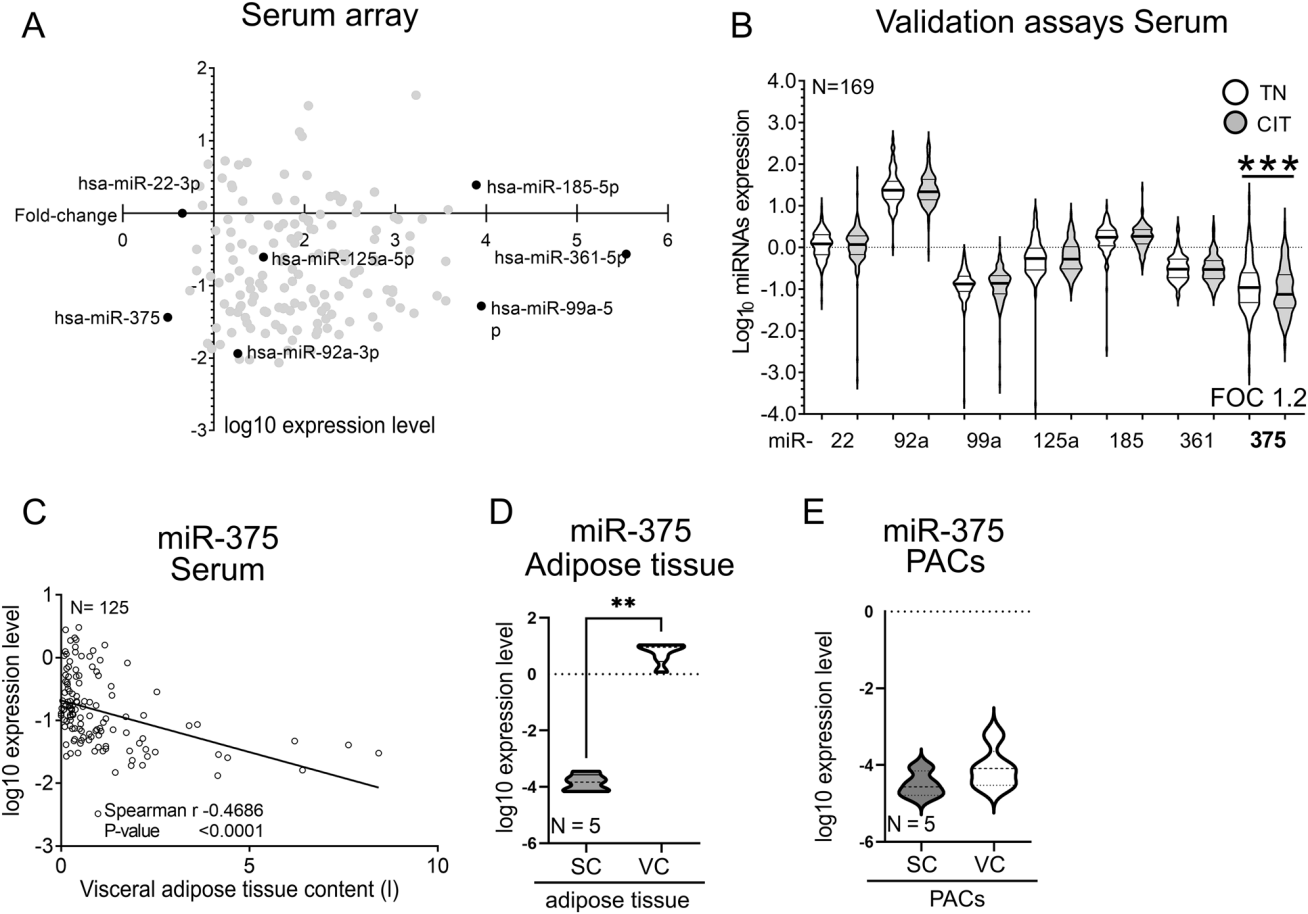
miRNA expression pattern in serum of healthy humans after acute cold exposure is visceral tissue associated. (**A**) Scatter plot analysis between logarithmic expression levels (C_q_) and fold-change of miRNAs after cold exposure measured via microarray. miRNAs chosen for validation analysis are labeled in black. (**B**) Serum samples analysis of 169 individuals undergoing cold exposure revealed significant downregulation of miR-375 (Wilcoxon signed-rank test, one-tailed, *P* ≤ *0.0001*). (**C**) Correlation of TN miR-375 levels with visceral adipose tissue content (Spearman r = − 0.469, *P* ≤ *0.0001*). (**D, E**) miR-375 expression levels in VC tissue and isolated pre-adipocytes in comparison to SC ones (Mann Whitney, one-tailed, *P* = *0.0022*). Data are normalized for relevant housekeeping genes, age and BMI adjusted, log_10_ transformed and shown as mean ± SEM. CIT = cold induced non-shivering thermogenesis, SC = subcutaneous, VC = visceral, TN = thermoneutral.

We profiled the miRNA spectra via the human miRCURY LNA miRNA Focus PCR Panels before and after cold exposure in pooled samples from 12 male participants to identify miRNAs affected by cold in serum. In a second array screening, we used pooled samples derived from 12 female individuals. (demographic data Table S1). We identified changes of 158 miRNAs in response to cold exposure in all samples, while miRNAs in males displayed stronger changes after cold exposure (FOC_males_ up to 5.5, FOC_females_ up to 1.8). Among the most abundant miRNAs in serum, five miRNAs including miR-22-3p, miR-99a-5p, miR-185-5p, miR-361-5p, and miR-375 were significantly changed after cold exposure in male samples (Fig. 1A). miR-361-5p displayed the most significant changes in the combined 12 sample by a 5.5-fold difference compared to the samples before CIT. Raw data of the array are shown in Table S4.

### Specific circulating miRNAs under CIT

To validate the five miRNAs identified in the discovery array, as well as miR-92a^14^ and miR-125^19,29^ which have been previously implicated in brown/beige adipocyte function, we used qPCR on an independent validation sample set comprising paired serum samples from 169 individuals before and after CE (demographic data Table S2). Among the selected miRNAs, miR-375 was predominantly down-regulated in the validation cohort (mean change − 0.922 to − 1.042, *p* ≤ 0.0001) (Fig. 1B).

### miR-375 is predominantly expressed in visceral adipose tissue and related to visceral adipose tissue mass

Correlating multiple clinical characteristics (see Table S5) with the miR-375 expression level, a moderate negative correlation of miR-375 levels under thermoneutral conditions with the visceral adipose tissue (VAT) volume was detected (r = − 0.47, *P* < 0.0001) (Fig. 1C). Analyzing miR-375 in human visceral and subcutaneous adipose tissue revealed a significantly higher expression level in VAT (Fig. 1D). Since SAT and VAT differ in cellular composition, expression differences of miR-375 between the two depots could also stem from non-adipogenic cells. Therefore, miR-375 expression was evaluated using isolated and in-vitro differentiated pre-adipocytes (PACs). Comparing miR-375 expression levels of VAT derived PACs in comparison to subcutaneous PACs a slightly higher level was detectable in visceral PACs, but did not reach significance (*p* = 0.2063) (Fig. 1E).

### MiR-375 target genes are involved in cold-induced thermogenesis

**Figure 2:**
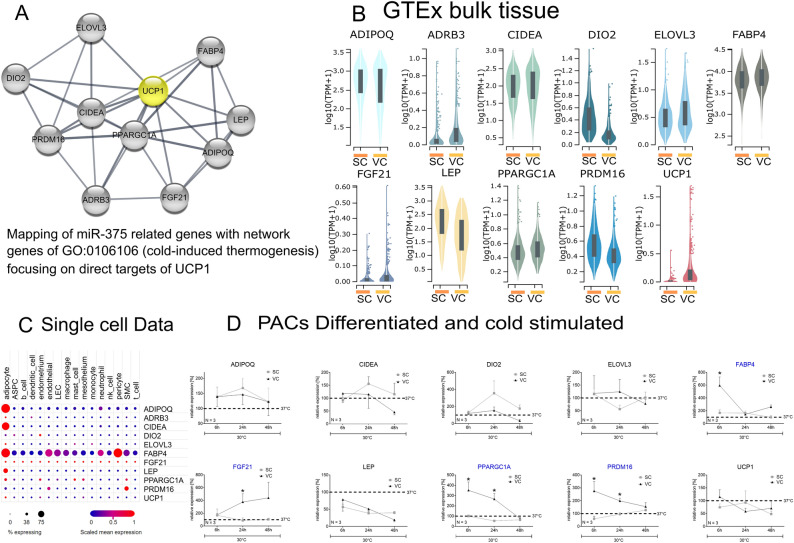
miR-375 related genes are thermogenesis associated and were differently regulated in VAT / SAT and cells. (**A**) Network of predicted miR-375 target genes involved in the adaptive cold induced-thermogenesis pathway, focusing on direct targets of UCP1. (**B**) Overall gene expression differences of the network genes between SAT and VAT adipose tissue, data originates from the “Genotype-Tissue Expression” project. (**C**) Using single-cell RNA read outs from the Single Cell Portal (study no. SCP137) gene expression levels of the network genes were displayed. (**D**) Comparison of PACs of different origin after acute cold stimulation revealed only for VC-PACs a response detectable in the up-regulation of the identified network genes *FABP4, FGF21, PPARGC1A* and *PRDM16* (*P < *0.05*). Data are normalized to relevant housekeeping genes and shown as mean ± SEM. SC = subcutaneous, VC = visceral.

Using in silico sequence-based target gene prediction, we identified numerous putative miR-375 target genes. Matching these target genes with those of the cold-induced thermogenesis pathway (GO:0106106) we were able to identify 144 overlapping genes. By using string.db and focusing on direct target genes related to uncoupling protein 1 (*UCP1*) we were able to create a network of direct interaction genes (interaction score = high confidence 0.7) (Fig. 2A). Namely, adiponectin (*ADIPOQ*), adrenoreceptor beta 3 (*ADRB3*), cell death activator (*CIDEA*), iodothyronine deiodinase 2 (*DIO2*), fatty acid elongase 3 (*ELOVL3*), fatty acid binding protein 4 (*FABP4*), fibroblast growth factor 21 (*FGF21*), leptin (*LEP*), peroxisome proliferator-activated receptor gamma coactivator 1-alpha (*PPARGC1A*) and PR/SET domain 16 (*PRDM16*) could be identified. The expression levels of the computationally derived genes were assessed for all following cell experiments.

### MiR-375 target genes are differentially expressed between VAT and SAT

To assess the overall gene expression differences between SAT and VAT we used data originating from the “Genotype-Tissue Expression (GTEx)” project. Briefly, GTEx is a public resource to study gene expression in various tissues and has been described elsewhere in detail^33,34^. Samples were harvested post-mortem with SAT originating from the leg and VAT from the greater omentum. From the identified miRNA-375 related genes and direct targets of UCP1 violin plots were generated using the GTEx portal’s “Multi Gene Query” (N-paired = 99, Fig. 2B). Gene expressions of *ADRB3, CIDEA, ELOVL3, FABP4* and *PPARGC1a* was higher in VAT comparied to SAT. Detailed fold changes are listed in Table S6. An opposite regulation was seen for *ADIPOQ, DIO2, LEP* and *PRDM16*. In addition, the thermogenesis-related genes *FGF21* and *UCP1* showed higher expression levels in VAT compared to SAT but overall expression remained at a low level.

### Adipocytes are the predominant cell type in WAT expressing miR-375 target genes

Using single-cell/nucleus RNA sequencing data publicly available on the Single Cell Portal (study no. SCP137) gene expression levels of the network genes in relation to WAT-resident cell types were displayed (Fig. 2C). The genes *ADIPOQ, CIDEA, FABP4, LEP* and *PPARGC1A* are predominantly found in adipocytes, as well as in adipocyte stem and precursor cells (Fig. 2C). Expression of *ADRB3, ELOVL3* and *UCP1* is solely detected in adipocytes but not in other cell types. The thyroid hormone activating gene *DIO2* is predominantly expressed in endometrium cells. *FABP4* as an ubiquitous fatty acid carrier protein is relevant in different cell types. The same applies for *FGF21*, which is important for cell survival and involved in a variety of biological processes. *PRDM16* was seen predominantly in smooth muscle cells (SMC), where it promotes muscle cell differentiation^35^. In white adipocytes, as used in this single cell data approach, only low expression of *PRDM16* could be expected because *PRDM16* specifies the brown fat lineage and is not involved in white adipogenesis. Regarding *UCP1*, only adipocytes did show detectable expression levels in comparison to the other cell types.

### Visceral pre-adipocytes respond to CE

To evaluate the cold response of paired SC and VC preadipocytes we exposed PACs to cold (30 °C) for several hours (Fig. 2D). Apoptosis as well as cell stress markers such as *p53* and lactatde hydrogenases (LDH) were measured over the time to ensure cellular integrity (Fig. S1). Both parameters showed no significant changes in comparison to control conditions. *ADRB3* expression was under the detection level in cells from both depots. *ADIPOQ, CIDEA, DIO2, ELOVL3, LEP* and *UCP1* gene expression did not show significant differences independent of the depot origin. However, gene expression of *FABP4* was significantly up-regulated in visceral PACs after 6 h of cold stimulation in comparison to subcutaneous PACs (*p* < 0.05). *FGF21* did show a significantly higher expression level (*p* < 0.05) for visceral PACs after 24 h of CE. Additionally, *PPARGC1A* and *PRDM16* showed a significantly stronger gene expression response for visceral PACs after 24 h of CE in comparison to subcutaneous PACs (*p* < 0.05). In general, the differentiated PACs of VAT origin showed a stronger response to cold stimulation. As our work indicates that miR-375 itself as well as its thermogenesis-related target genes act primarily in VAT, visceral PACs were used for all following up experiments.

### miR-375 knock down in visceral PACs elevates important adipogenesis and thermogenesis genes

**Figure 3:**
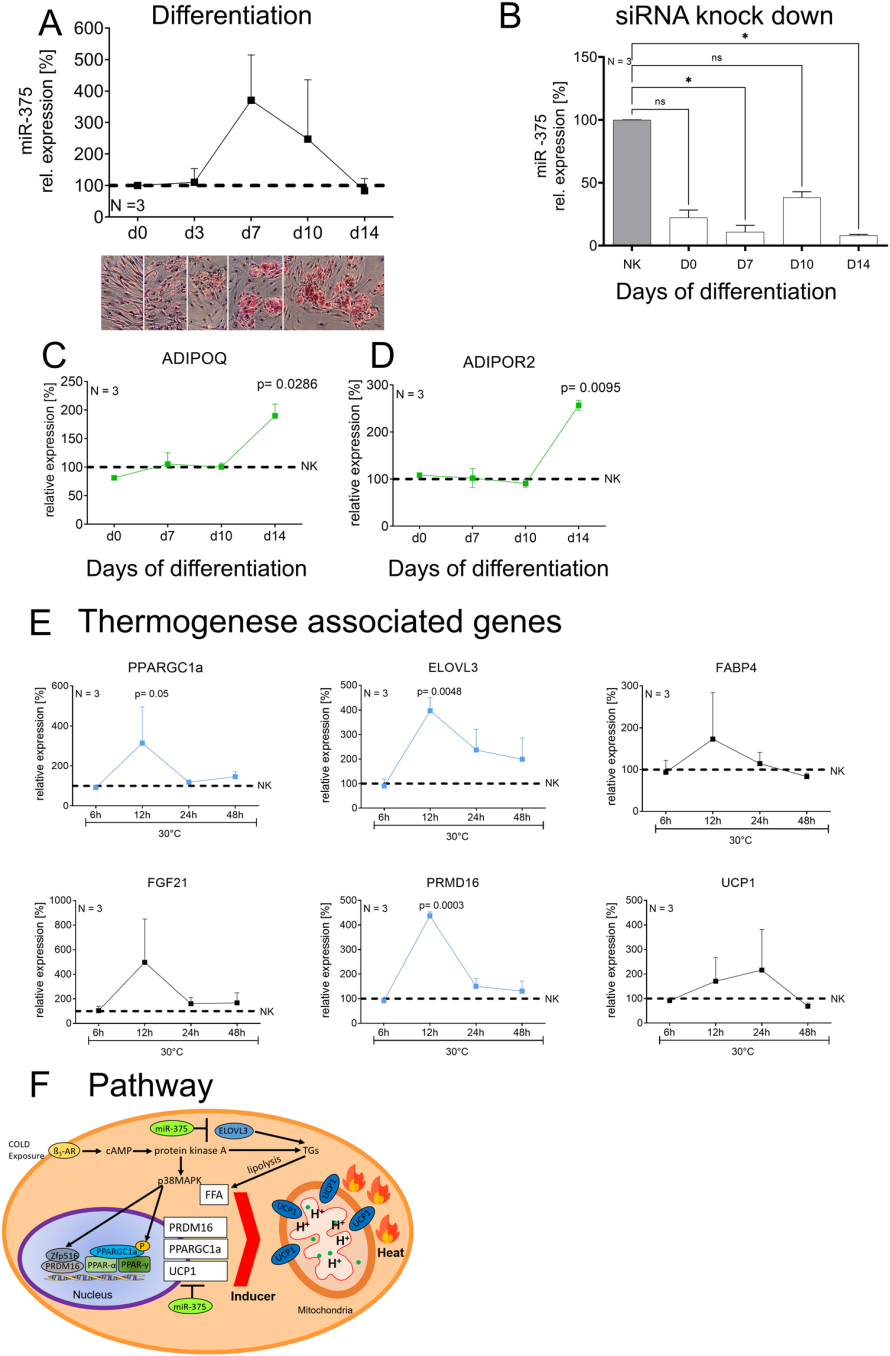
Depletion of miR-375 in VC-PACs led to increased thermogenesis genes under cold stimulation. (**A**) Relative miR-375 gene expression over the differentiation period in VC-PACs. Corresponding Oil Red O staining is displayed below. (**B**) siRNA knock down of miR-375 at day 0, 7, 10 and day 14 of adipogenic differentiation of VC-PACs. (**C, D**) Depletion of miR-375 elevated ADIPOQ and ADIPOR2 gene expression in fully differentiated VC-PACs. (**E**) Corresponding gene analyses showed significant changes of *PPARGC1A, ELOVL3* and *PRDM16 i*n cold stimulated miR-375 knock down cells of VC adipose tissue. (**F**) Associated adipogenic/beiging pathway influenced by miR-375. Data are normalized to relevant housekeeping genes and shown as mean ± SEM. Statistical significance were defined as follows: **P < *0.01*, ***P < *0.001*. SC = subcutaneous, VC = visceral.

To understand the influence of miR-375 on the differentiation process of adipocytes we first investigated its expression level on day 0, 3, 7, 10 and 14 of differentiation in visceral PACs (Fig. 3A). In parallel, the differentiation state of the cells was tested by oil red staining. During adipogenic differentiation, we observed increasing levels of miR-375 gene expression in in vitro cultured human pre-adipocytes until day 7 followed by a decrease until day 14 (Fig. 3A). Transfection of the cells with siRNA-miR-375 three days before the induction for differentiation revealed a high knock-down efficiency of up to 89% in comparison to the mock transfection (NK) siRNA (Fig. 3B). The knock down remained stable up to d14 of the differentiation process (61–89% efficiency). In parallel with the knockdown of miR-375, we measured the adipogenesis marker genes *ADIPOQ* and *ADIPOR2*. Both genes were significantly up-regulated in fully differentiated VC-PACs on d14 when miR-375 was repressed (*p* = 0.0286, *p* = 0.001, Fig. 3C,D).

Because of the strong response to cold exposure between 6 and 24 h of visceral PACs in the first experiment (Fig. 2D), we decided to harvest transfected and differentiated VC-PACs after 6 h, 12 h, 24 h and 48 h at 30 °C, respectively. Analyzing gene expression after 12 h of cold exposure revealed significantly higher expression levels of *PPARGC1A, ELOVL3*, and *PRDM16* in VC-PACs (Fig. 3E). Additionally, higher levels of *FABP4* and *FGF21* were detected but did not reach significance.

Summarizing, cold exposure of volunteers had a suppressing effect on miR-375 in serum. As miR-375 is predominantly found in VAT, this finding implicated a specific function of this miRNA for this adipose tissue depot. Cold stimulation (in vitro) affected gene expression primarily in PACs derived from the visceral depot. Therefore, visceral PACs were used in all follow up experiments to study miR-375 related molecular thermogenesis questions. miR-375 inhibition in visceral PACs induced important thermogenesis genes, indicating that miR-375 is involved in browning/beigeing as well as adipogenic processes in cells of visceral origin (Fig. 3F). The up-regulation of important adipogenesis and thermogenesis genes raises the possibility that suppression of miR-375 is related to cell fate processes and supports thermogenesis.

## Discussion

Cold-induced non-shivering thermogenesis received growing interest, as studies in humans suggested that the underlying activation of BAT could play a significant role in energy homeostasis^13^. This study aimed at identifying miRNAs that are modulated by cold exposure. Out of the seven miRNAs screened, miR-375 expression was negatively associated with the cold response in the participants. Since their discovery in 1993^36^, miRNAs have been widely studied concerning their potential use for diagnostic and prognostic purposes. Specific circulating miRNA profiles have been reported to play a role in various diseases including cancer^37^, osteoporosis, skeletal-associated diseases^38,39^, coronary heart disease^40,41^, and metabolic diseases such as obesity^42^ and type 2 diabetes^43^. Here, we report for the first time a serum-based miRNA profiling following a defined cold exposure as part of a highly standardized human intervention study.

We detected a downregulation of miR-375 under cold exposure in serum and an inverse correlation to VAT volume, suggesting a strong involvement of this miRNA in visceral adipose tissue homeostasis. These findings were underlined by the high expression of miR-375 in visceral adipose tissue and higher expression in visceral preadipocytes in comparison to subcutaneous PACs. *Hu, *et al*.* identified miR-375 as a “marker” to distinguish the different fat depot-derived stem cells and showed that miR-375 was most abundantly expressed in visceral PACs^44^. Our target enrichment analysis provided a specific link between miR-375 and cold-induced thermogenesis, suggesting that miR-375 may have a generic role on diverse cold-induced processes in human adipose tissue. *In-silico* predicted target genes of the thermogenesis pathway were predominantly regulated in visceral adipose tissue and mainly connected to cells of adipose tissue origin^15,34,45^. VAT is known to have a higher secretory capacity than SAT, and this difference is an intrinsic feature of its cellular components^46^. On a cellular level, cold exposure experiments revealed a stronger reaction of visceral PACs on key genes involved in the miR-375 thermogenesis pathway, including *FABP4, FGF21, PPARGC1A* and *PRDM16*.

Compared to subcutaneous adipocytes, visceral adipocytes show higher fatty acid turnover and lipolysis rates. Furthermore, mature visceral adipocytes are less responsive to the antilipolytic effect of insulin^47^. Additionally, recent omics studies suggest a higher content of cells with the potential to perform thermogenesis in VAT compared to SAT^14,15,48^. Both, the greater lipolytic capacity and involvement in browning-related processes indicate a high relevance of VAT for CIT.

To date, some studies have indicated that specific miRNAs are involved in BAT activation upon cold exposure^49,50^. However, these studies mainly focused on identifying miRNAs in one cell type, such as immortalized brown adipocytes or murine mesenchymal stem cells. Also, a regulatory effect of certain miRNAs in specific animal models such as *Ercc1*^−*/*−^, *Dgcr8flox/flox*, adiponectin-Cre transgenic mice, or *Pparα*^−*/*−^ was described^51^. For example, *Oliverio *et al*.* could identify miR-328 as a controller of brown adipose tissue differentiation and BAT function in mice^25^. In human individuals undergoing a mild cold exposure, only exosomal expression changes of miR-92a and miR-122-5p were reported^19,52^. Both groups found negative associations of these exo-miRNAs and BAT activation in mice and humans. In contrast, we analyzed circulating miRNAs because exosomal miRNAs are mainly detected upon malignant conditions. Further, the majority of exo-miRs is not conserved in exosomes but rather bound to argonaut proteins and is therefore not captured^53^. Further, by analyzing exo-miRs, the spectrum of detectable miRNAs is lower^54^. In the present study, miR-375 was the only circulating miRNA that significantly changed after cold exposure in both the exploratory screening and validation cohort. In a study in individuals with or without diabetes, an up-regulation of miR-375 was detected in the diabetic group^55^. miR-375 has been shown to promote adipogenic differentiation by increasing mRNA levels of *C/EBP/α* and *PPARG2* as well as by inducing adipocyte fatty acid-binding protein and triglyceride accumulation^56^. In addition, miR-375 is upregulated during the osteogenic differentiation of human adipose-derived mesenchymal stem cells^57,58^ highlighting its impact on mesenchymal cell differentiation. *Kraus *et al*.* identified miR-375 as an androgen-regulated miRNA, showing an androgen-mediated inhibition of miR-375 and the associated regulation of *ADIPOR2* in differentiating human adipocytes^59^. We could confirm these findings by miR-375 knock out revealing an up-regulation of *ADIPOR2* and *ADIPOQ* in visceral PACs. Cold exposure experiments in visceral PACs revealed significantly higher expression levels of the thermogenesis genes *PPARGC1A*, *ELOVL3,* and *PRDM16*. An up-regulation of *PPARGC1A* is known to promote thermogenesis in adipose tissue and skeletal muscle^60^. In brown adipocytes, the transcriptional coactivator *PPARGC1A* is a key regulator of *UCP1* expression, mitochondrial biogenesis and oxidative metabolism^61^. *ELOVL3* elongates very long chain fatty acid (VLCFA) in adipose tissue. The suggested function in cold-stimulated BAT is to replenish intracellular pools of specific VLCFAs when the fatty acid turnover rate is high^62^. The gene *PRDM16* is a powerful driver of brown and beige adipocyte identity^63,64^. *PRDM16* co-activates *PPARγ* and *PPARα* in adipocytes to activate the expression of thermogenic genes, like *UCP1*. miRNA-133 was found to be markedly downregulated in BAT and SAT of C57Bl/6N mice and negatively regulates *PRDM16* as well^65^. In mice exposed to cold, levels of miR-33 in BAT were rapidly downregulated, consistent with a role for miR-33 in repressing adaptive thermogenesis^66^. Mainly, miRNAs have been shown to be potent repressors of brown fat differentiation by targeting different transcriptional coactivators.

It is widely accepted that miRNAs have multiple—sometimes hundreds—of targets. This supports our findings of the depletion of miR-375, which might have multi-effects on adipocyte maturation as well as on thermogenic pathways. miR-375 and several other miRNAs have been identified to exert prominent roles in regulating the beiging of inguinal white AT and activation of BAT^67^.

In conclusion, our findings raise interesting questions about the role of miRNA-375 in the beiging/browning process of adipocytes and underlines the need to understand the biological origins and functions of miRNAs in this context. We could show a decreased expression of miR-375 after cold stimulation in humans. Tissue and cell comparison identified a prominent role of miR-375 in the visceral fat depot. Depletion of miR-375 revealed interactions with cell fate and thermogenic response in visceral adipose tissue.

## Methods

### Study approval

The ethical review committee of the Faculty of Medicine of the Technical University of Munich approved the FREECE (Effect of the FTO-Genotype on Resting Energy Expenditure after defined Cold Exposure) study protocol (project number 236/16) as well as the MOBB study (Munich obesity biobank, project number 5716/13). All participants provided written informed consent before any study procedures. All procedures were conducted according to the principles of the Declaration of Helsinki. The demographic data of the included individuals are presented in the supplemental material (Tables S1, S2 and S3).

### Short-term cold exposure

All participants underwent the procedure in the morning and in the fasted state. First, anthropometric measurements were taken and body composition was assessed through bio impedance analysis (TANITA Body Composition Analyzer Type BC-418 MA, Tanita Europe GmbH, Sindelfingen, Germany). Blood pressure and heart rate were measured and skin temperature was recorded using iButtons (Thermochron, Wisconsin, United States). Eight sensors were placed on defined sites of the body to assess the overall skin temperature according to ISO 988620^68^. One additional iButton was placed at the site of the supraclavicular area. The participants were placed in a supine position and allowed to rest for 15 min, followed by 30 min of measurement of resting energy expenditure by indirect calorimetry (Cosmed Quark RMR 1.0, Fridolfing, Germany). Blood was drawn from each participant, followed by the subsequent non-shivering cold exposure over two hours using water-containing thermic blankets (Maxi-Therm Lite, Cincinnati Sub-Zero Products, LLC). In the last 30 min of cold exposure, resting metabolic rate was measured again. Thereafter, the second blood drawing took place. A detailed description of the cooling protocol was recently published^31^.

### Blood sample analysis

Serum samples were analyzed by a certified laboratory (Synlab, Munich, Germany) for free triiodothyronine (T_3_), C-reactive protein, and triglycerides. Non-esterified fatty acids (Wako Chemicals, Neuss, Germany), insulin (DRG Instruments, Marburg, Germany), leptin, and total adiponectin (R&D, Wiesbaden, Germany) were analyzed in plasma using commercially available ELISA kits according to the instructions of the manufacturers.

### GTEx and single cell data

Violin plots displaying log10 transcripts per million + 1 (TPM + 1) of miR-375 related genes were generated using the Genotype-Tissue Expression (GTEx) project’s multi gene query function (Release V8)^33,34^. Dotplots of white adipose tissue single-cell RNA expression were retrieved from the Single Cell Portal (study no. SCP1376)^15^.

### Isolation, culture and differentiation of human preadipocytes (PAC)

All methods for the isolation, culture and differentiation of human PACs derived from adipose tissue material are described in detail, elsewhere^69,70^. Briefly, PACs were isolated from adipose tissue material based on collagenase-digestion, grown until confluency in T-75 flasks (Falcon, Corning, NY) and cryopreserved. For differentiation experiments cells were thawed, grown until confluency in T-25 flasks (Falcon) and splitted on to 6-well plates (Falcon). At confluency, differentiation was induced and cells were cultured for 14 days to allow for differentiation and accumulation of lipids. Cells were cold stimulated up to 48 h at 30 °C. H&E and Oil red O staining, LDH activity measurement and RNA isolation were performed using established methods, respectively.

### siRNA transfection

For transfection of anti-miR-375 oligonucleotides, visceral PACs were seeded on 6-well plates. At a density of 80%, medium was changed and cells were transfected with miRCURY LNA miR-375 Inhibitor or Inhibitor Control (Qiagen, Hilden, Germany) for 72 h with a final concentration of 30 nM. Cells were differentiated up to 14 days, followed by cold exposure experiments. RNA and supernatants were harvested on d0, d3, d7, d10 and d14. Cold exposure of the cells was carried out for 6 h, 12 h, 24 h and 48 h. Differences in expression level of miR-375 related genes were analyzed by RT-qPCR.

### mRNA/miRNA extraction

Serum samples frozen at − 80 °C were thawed on ice and centrifuged at 16,000×*g* for 5 min at 4 °C. miRNA was extracted from 200 µl serum using TRIzol™ Reagent (Thermo Fisher Scientific, Massachusetts, USA) and the miRNeasy Serum/Plasma Advanced Kit, according to the recommendations of the manufacturer (Qiagen, Hilden, Germany). RNA was precipitated with 900µI ethanol, triple washed with wash solution, followed by RNA elution in 20 μl nuclease-free water and stored at − 80 °C. Regarding cells, total RNA including microRNAs was isolated at several time points using TRIzol™ Reagent (Thermo Fisher Scientific, Massachusetts, USA). The amount and integrity of isolated miRNA was evaluated via gel electrophoresis using a Bioanalyzer device (Small Chip, Agilent Technologies, California, USA). Serum samples were analyzed regarding their hemolytic status. Therefore, miR-451, a miRNA highly abundant in red blood cells was used. The concentration of miR-451 was significantly lower in all samples compared to our internal positive control (Cutoff Cq value = 15).

### qPCR analysis

The isolated miRNA as well as the spike-in control UniSp6 to assess enzyme efficiency were reversely transcribed into cDNA using the miRCURY LNA RT Kit (Qiagen, Hilden, Germany). The reaction was incubated at 42 °C for 60 min and then heat-inactivated at 95 °C for 5 min. 20-fold diluted cDNA samples were stored at − 20 °C. Real-Time quantitative PCR (RT-qPCR) was carried out using custom 384 well panels (4titude, Wotton, UK). For RT-qPCR analysis, each reaction contained 4 µl of cDNA and 6 µl of master mix using miRCURY Sybr Green Kit and LNA-enhanced miRNA primer assays (Qiagen, Hilden, Germany). PCR conditions were 95 °C for 2 min, 50 cycles of denaturation (95 °C, 10 s) and annealing (56 °C, 60 s), and a melting curve analysis to complete the run on a LC480 Real-Time PCR system (Roche, Basel, Switzerland). SNORD38b and SNORD48b served as internal control for tissue and cells, let-7i-5p and miR-30e-5p for serum.

For analyzing the expression levels of miRNA-375 related genes, cDNA was synthesized from 1 mg total RNA using High capacity cDNA RT Kit (Applied Biosystems, Germany). RT-PCR was performed with 20 ng cDNA with the Maxima SYBR Green Master Mix (ThermoScientific, Germany) using specific primer pairs (Table S6). For normalization, GAPDH and IPO8 were used. To calculate the C_q_-values, the second derivative method was used.

### miRNA array analysis

We profiled miRNA spectra from two pooled serum groups, including one pool of 12 males and another of 12 female samples, to identify regulated miRNAs during cold-induced thermogenesis. In total, 169 different miRNAs were profiled by the human miRCURY LNA miRNA Focus PCR Panels YAHS-106YG-2 (Qiagen, Hilden, Germany). The cycle number via RT-qPCR determined the expression levels. Levels were normalized to the internal reference genes using the 2^−∆∆Ct^ method^71^. Afterwards, the fold-change before and after cold exposure regarding the expression of specific miRNAs was calculated.

### Cell staining

Cells were fixed with 4% paraformaldehyde for 20 min at room temperature. After one washing step with PBS, Oil Red O solution was added for 60 min and rinsed off with PBS until clear. For a better contrast, cells were counterstained with HE. After one washing step with PBS, hematoxylin solution (VWR, Heamalum (Mayer´s) Gurr for microscopy) was added for 4 min and rinsed off with PBS until clear. Eosin solution (2% in acetic EtOH) were added for 2 min. After rinsing with PBS stained cells were assessed under a light microscope (VHX series, Keyence, Osaka, Japan).

### Measurement of lactate dehydrogenase activity

Lactate dehydrogenase (LDH) release in the incubation medium was measured as an index for cytotoxicity. As control stimulated medium at 30 °C for up to 48 h were used. Aliquots of the incubation medium were added to an assay mixture containing 81 nM Tris, 203 nM NaCl, and 0.24 nM NADH, pH 7.2. The reactions were started by the addition of 1.55 mM pyruvate, and the conversion of NADH into NAD^+^ was measured spectrophotometrically at 340 nm at 30 °C for 30 min. Results were compared to the control (medium only).

### Bioinformatics predictions

To identify human miR-375-regulated genes, we first employed the miRNA search tool MultiMiR in R, compiling nearly 50 million records from 14 different databases, including miRTarBase, TarBase, miRanda, miRDB and Targetscan^72,73^. We then investigated the functional interaction between the lists of predicted miR-375 target genes with a comprehensive list of genes associated with cold exposure (gene ontology term 0106106). To generate a map of miR-375 target genes involved in CIT the String DB tool and the program cytoscape were used^74^. Afterwards we filtered genes, which were direct targets of UCP1, as this is the central gene of the thermogenesis pathway.

### Statistical analyses

Results are given (after age and BMI adjustment as well as logarithmic transformation) as violin plots, mean with standard error of the mean (± SEM). All data were non-Gaussian distributed (Kolmogorov–Smirnov test *P* < *0.05*). Two-tailed unpaired Mann Whitney test were used to determine the significance of group-wise differences. GraphPad Prism version 9 was used (Graph Pad Software, San Diego, USA). *P-values* < *0.05* were considered significant.

## Supplementary Information

Supplementary Information.
