# Supplementary material for: miR-375 is cold exposure sensitive and drives thermogenesis in visceral adipose tissue derived stem cells
Source: Sci Rep. 2022 Jun 10;12:9557. doi: 10.1038/s41598-022-13610-6 (PMC9187663; doi:10.1038/s41598-022-13610-6)

**Supplemental material**

**miR-375 is cold exposure sensitive and drives thermogenesis in visceral adipose tissue derived stem cells**

**Table S1:** Demographical characteristics of the donors of the samples used in the array

|  | Male | Female |
| --- | --- | --- |
| patients (N) | 12 | 12 |
| Age (y), mean (range) | 28.0 (23-39) | 26.0 (21-30) |
| Body mass index (kg/m2) mean, ± SEM | 23.2 ± 0.4 | 21.7 ± 0.6 |

**Table S2:** Demographical characteristics of the donors of the serum samples used in the validation assays

|  | Individuals |
| --- | --- |
| patients (N) | 169 |
| Age (y), mean (range) | 26.1 (18-52) |
| Gender (f:m) | 97:72 |
| Body mass index (kg/m2) mean, ± SEM | 24.6 ± 0.4 |

**Table S3:** Demographical characteristics of the donors of the adipose tissue used for

cell culture experiments

|  | Individuals |
| --- | --- |
| included patients (N) | 3 |
| Age (y), mean (range) | 38.3 (31-48) |
| Gender (f:m) | 2:1 |
| Body mass index (kg/m2) mean, ± SEM | 52.73 ± 4.3 |

**Table S4:** Analyzed data of the pooled samples (female/ male) using the Human Serum/Plasma Focus, miRCURY LNA miRNA Focus PCR Panel. Data were normalized to the internal housekeeping miRNA genes of the array using the 2^-∆∆Cq^ method. AC= after cold exposure, BC= before cold exposure, FOC = fold of change

|  | Male | | | Female | | |
| --- | --- | --- | --- | --- | --- | --- |
| miRNA | BC | AC | FOC | BC | AC | FOC |
| hsa-let-7a-5p | 0.375 | 0.765 | 2.042 | 0.519 | 0.537 | 1.035 |
| hsa-let-7b-3p | 0.018 | 0.026 | 1.404 | 0.026 | 0.019 | 0.717 |
| hsa-let-7b-5p | 1.458 | 1.278 | 0.877 | 0.843 | 0.923 | 1.094 |
| hsa-let-7c-5p | 0.251 | 0.826 | 3.294 | 0.484 | 0.564 | 1.165 |
| hsa-let-7d-3p | 0.111 | 0.272 | 2.462 | 0.148 | 0.175 | 1.181 |
| hsa-let-7d-5p | 0.158 | 0.436 | 2.770 | 0.355 | 0.481 | 1.357 |
| hsa-let-7e-5p | 0.127 | 0.274 | 2.158 | 0.209 | 0.256 | 1.223 |
| hsa-let-7f-5p | 0.014 | 0.015 | 1.072 | 0.018 | 0.017 | 0.953 |
| hsa-let-7g-5p | 0.284 | 0.596 | 2.099 | 0.494 | 0.755 | 1.526 |
| hsa-let-7i-5p | 0.443 | 0.962 | 2.173 | 1.113 | 0.982 | 0.883 |
| hsa-miR-1 | N.A. | 0.007 | N.A. | N.A. | N.A. | N.A. |
| hsa-miR-7-1-3p | N.A. | 0.014 | N.A. | N.A. | N.A. | N.A. |
| hsa-miR-7-5p | 0.029 | 0.037 | 1.266 | 0.063 | 0.045 | 0.712 |
| hsa-miR-10b-5p | 0.058 | 0.149 | 2.549 | 0.093 | 0.083 | 0.895 |
| hsa-miR-15a-5p | 0.891 | 1.520 | 1.705 | 1.530 | 1.574 | 1.028 |
| hsa-miR-15b-3p | 0.030 | 0.072 | 2.378 | 0.041 | 0.064 | 1.548 |
| hsa-miR-15b-5p | 0.605 | 0.843 | 1.395 | 0.765 | 0.719 | 0.940 |
| hsa-miR-16-2-3p | 0.042 | 0.060 | 1.424 | 0.053 | 0.075 | 1.414 |
| hsa-miR-16-5p | 14.867 | 30.358 | 2.042 | 19.481 | 24.830 | 1.275 |
| hsa-miR-17-5p | 0.744 | 1.698 | 2.282 | 1.438 | 1.389 | 0.966 |
| hsa-miR-18a-5p | 0.112 | 0.271 | 2.412 | 0.232 | 0.204 | 0.877 |
| hsa-miR-18b-5p | 0.061 | 0.093 | 1.537 | 0.096 | 0.101 | 1.050 |
| hsa-miR-19a-3p | 3.640 | 4.640 | 1.275 | 4.420 | 4.482 | 1.014 |
| hsa-miR-19b-3p | 5.042 | 4.737 | 0.940 | 4.904 | 4.153 | 0.847 |
| hsa-miR-20a-5p | 2.778 | 3.040 | 1.094 | 2.352 | 2.574 | 1.094 |
| hsa-miR-20b-5p | 0.042 | 0.105 | 2.479 | 0.094 | 0.092 | 0.979 |
| hsa-miR-21-5p | 3.665 | 5.007 | 1.366 | 3.147 | 3.019 | 0.959 |
| **hsa-miR-22-3p** | **1.520** | **0.996** | **0.655** | **1.235** | **1.105** | **0.895** |
| hsa-miR-22-5p | 0.014 | 0.022 | 1.569 | 0.045 | 0.022 | 0.486 |
| hsa-miR-23b-3p | 0.704 | 1.951 | 2.770 | 1.342 | 1.201 | 0.895 |
| hsa-miR-24-3p | 1.951 | 3.444 | 1.765 | 1.201 | 2.683 | 2.235 |
| hsa-miR-25-3p | 4.672 | 5.256 | 1.125 | 2.241 | 3.061 | 1.366 |
| hsa-miR-26a-5p | 0.749 | 1.758 | 2.346 | 1.408 | 1.520 | 1.079 |
| hsa-miR-26b-5p | 0.404 | 0.714 | 1.765 | 0.584 | 0.749 | 1.283 |
| hsa-miR-27a-3p | 0.605 | 1.448 | 2.395 | 1.113 | 1.105 | 0.993 |
| hsa-miR-27b-3p | 0.271 | 0.820 | 3.031 | 0.560 | 0.537 | 0.959 |
| hsa-miR-28-3p | 0.029 | 0.078 | 2.694 | 0.045 | 0.071 | 1.580 |
| hsa-miR-28-5p | 0.017 | 0.062 | 3.555 | 0.043 | 0.051 | 1.165 |
| hsa-miR-29a-3p | 0.288 | 0.377 | 1.310 | 0.352 | 0.288 | 0.818 |
| hsa-miR-29b-3p | 0.020 | 0.045 | 2.189 | 0.026 | 0.037 | 1.395 |
| hsa-miR-29c-3p | 0.560 | 0.572 | 1.021 | 0.609 | 0.657 | 1.079 |
| hsa-miR-30a-5p | 0.063 | 0.163 | 2.603 | 0.124 | 0.124 | 0.993 |
| hsa-miR-30b-5p | 0.458 | 0.545 | 1.189 | 0.385 | 0.622 | 1.613 |
| *Continued from page 2* | | | | | | |
| hsa-miR-30c-5p | 0.292 | 0.605 | 2.071 | 0.263 | 0.370 | 1.404 |
| hsa-miR-30d-5p | 0.396 | 1.017 | 2.567 | 0.605 | 0.787 | 1.301 |
| hsa-miR-30e-3p | 0.016 | 0.035 | 2.235 | 0.023 | 0.026 | 1.125 |
| hsa-miR-30e-5p | 0.294 | 0.879 | 2.990 | 0.600 | 0.635 | 1.057 |
| hsa-miR-32-5p | 0.028 | 0.044 | 1.558 | 0.073 | 0.065 | 0.889 |
| hsa-miR-33a-5p | 0.027 | 0.043 | 1.580 | 0.078 | 0.078 | 1.000 |
| hsa-miR-34a-5p | 0.008 | 0.010 | 1.283 | 0.012 | 0.023 | 2.028 |
| **hsa-miR-92a-3p** | **9.088** | **11.503** | **1.266** | **8.901** | **10.296** | **1.157** |
| hsa-miR-92b-3p | 0.010 | 0.012 | 1.117 | 0.011 | 0.013 | 1.149 |
| hsa-miR-93-3p | 0.058 | 0.056 | 0.966 | 0.032 | 0.019 | 0.582 |
| **hsa-miR-99a-5p** | **0.034** | **0.134** | **3.945** | **0.074** | **0.082** | **1.117** |
| hsa-miR-99b-5p | 0.027 | 0.052 | 1.972 | 0.058 | 0.045 | 0.785 |
| hsa-miR-101-3p | 1.113 | 1.468 | 1.320 | 0.996 | 1.017 | 1.021 |
| hsa-miR-106a-5p | 0.982 | 1.663 | 1.693 | 1.541 | 1.489 | 0.966 |
| hsa-miR-106b-3p | 0.013 | 0.024 | 1.919 | 0.017 | 0.011 | 0.683 |
| hsa-miR-106b-5p | 0.501 | 1.296 | 2.585 | 0.787 | 1.038 | 1.320 |
| hsa-miR-107 | 0.652 | 1.574 | 2.412 | 1.287 | 1.489 | 1.157 |
| hsa-miR-122-5p | 0.709 | 0.820 | 1.157 | 0.765 | 0.630 | 0.824 |
| **hsa-miR-125a-5p** | **0.160** | **0.247** | **1.548** | **0.184** | **0.190** | **1.035** |
| hsa-miR-125b-5p | 0.082 | 0.226 | 2.770 | 0.140 | 0.122 | 0.871 |
| hsa-miR-126-3p | 2.034 | 2.435 | 1.197 | 1.845 | 2.105 | 1.141 |
| hsa-miR-126-5p | 0.391 | 0.317 | 0.812 | 0.284 | 0.231 | 0.812 |
| hsa-miR-127-3p | 0.005 | 0.011 | 2.362 | 0.014 | 0.017 | 1.197 |
| hsa-miR-128-3p | 0.017 | 0.029 | 1.682 | 0.051 | 0.035 | 0.688 |
| hsa-miR-130a-3p | 0.137 | 0.244 | 1.778 | 0.372 | 0.402 | 1.079 |
| hsa-miR-130b-3p | 0.027 | 0.036 | 1.347 | 0.045 | 0.048 | 1.057 |
| hsa-miR-132-3p | 0.013 | 0.032 | 2.497 | 0.022 | 0.025 | 1.149 |
| hsa-miR-133a-3p | 0.018 | 0.040 | 2.297 | 0.071 | 0.038 | 0.525 |
| hsa-miR-133b | N.A. | 0.014 | N.A. | N.A. | 0.013 | N.A. |
| hsa-miR-136-3p | N.A. | 0.007 | N.A. | N.A. | 0.011 | N.A. |
| hsa-miR-136-5p | 0.005 | 0.021 | 4.028 | 0.011 | 0.022 | 2.014 |
| hsa-miR-139-5p | 0.014 | 0.030 | 2.144 | 0.035 | 0.037 | 1.072 |
| hsa-miR-140-3p | 0.428 | 0.685 | 1.602 | 0.505 | 0.541 | 1.072 |
| hsa-miR-140-5p | 0.049 | 0.071 | 1.444 | 0.043 | 0.037 | 0.865 |
| hsa-miR-141-3p | 0.007 | 0.010 | 1.395 | 0.010 | 0.002 | 0.193 |
| hsa-miR-142-3p | 1.858 | 2.241 | 1.206 | 2.385 | 1.992 | 0.835 |
| hsa-miR-143-3p | 0.061 | 0.217 | 3.555 | 0.130 | 0.121 | 0.933 |
| hsa-miR-144-3p | 2.778 | 2.352 | 0.847 | 4.124 | 3.742 | 0.908 |
| hsa-miR-144-5p | 0.030 | 0.057 | 1.892 | 0.036 | 0.083 | 2.329 |
| hsa-miR-145-5p | 0.153 | 0.484 | 3.160 | 0.765 | 0.605 | 0.790 |
| hsa-miR-146a-5p | 0.251 | 0.484 | 1.932 | 0.385 | 0.422 | 1.094 |
| hsa-miR-146b-5p | 0.021 | 0.072 | 3.458 | 0.018 | 0.065 | 3.555 |
| hsa-miR-148a-3p | 0.121 | 0.280 | 2.313 | 0.242 | 0.195 | 0.807 |
| hsa-miR-148b-3p | 0.198 | 0.161 | 0.812 | 0.127 | 0.181 | 1.424 |
| hsa-miR-150-5p | 1.640 | 3.326 | 2.028 | 1.629 | 2.164 | 1.329 |
| hsa-miR-148b-3p | 0.198 | 0.161 | 0.812 | 0.127 | 0.181 | 1.424 |
| hsa-miR-150-5p | 1.640 | 3.326 | 2.028 | 1.629 | 2.164 | 1.329 |
| hsa-miR-151a-3p | 0.125 | 0.218 | 1.741 | 0.103 | 0.153 | 1.495 |
| *Continued from page 2* | | | | | | |
| hsa-miR-151a-5p | 0.186 | 0.226 | 1.214 | 0.274 | 0.242 | 0.883 |
| hsa-miR-151a-5p | 0.186 | 0.226 | 1.214 | 0.274 | 0.242 | 0.883 |
| hsa-miR-152-3p | 0.074 | 0.143 | 1.932 | 0.185 | 0.127 | 0.688 |
| hsa-miR-154-5p | 0.007 | 0.013 | 1.815 | 0.013 | 0.008 | 0.620 |
| hsa-miR-155-5p | 0.014 | 0.013 | 0.979 | 0.012 | 0.012 | 0.979 |
| hsa-miR-181a-5p | 0.090 | 0.220 | 2.445 | 0.140 | 0.260 | 1.853 |
| **hsa-miR-185-5p** | **0.630** | **2.452** | **3.891** | **1.113** | **1.323** | **1.189** |
| hsa-miR-186-5p | 0.090 | 0.138 | 1.537 | 0.122 | 0.107 | 0.877 |
| hsa-miR-192-5p | 0.124 | 0.160 | 1.283 | 0.132 | 0.142 | 1.072 |
| hsa-miR-193a-5p | 0.044 | 0.090 | 2.071 | 0.047 | 0.057 | 1.223 |
| hsa-miR-194-5p | 0.082 | 0.114 | 1.395 | 0.119 | 0.140 | 1.173 |
| hsa-miR-195-5p | 0.005 | 0.009 | 1.866 | 0.013 | 0.013 | 1.050 |
| hsa-miR-197-3p | 0.208 | 0.580 | 2.789 | 0.184 | 0.315 | 1.717 |
| hsa-miR-199a-3p | 0.204 | 0.385 | 1.892 | 0.471 | 0.304 | 0.646 |
| hsa-miR-199a-5p | 0.064 | 0.090 | 1.404 | 0.162 | 0.148 | 0.914 |
| hsa-miR-200a-3p | N.A | N.A | N.A | N.A | N.A | N.A |
| hsa-miR-200c-3p | 0.010 | 0.021 | 2.129 | 0.007 | 0.013 | 2.014 |
| hsa-miR-205-5p | 0.020 | 0.059 | 2.949 | 0.042 | 0.052 | 1.257 |
| hsa-miR-208a-3p | N.A | N.A | N.A | N.A | N.A | N.A |
| hsa-miR-210-3p | 0.024 | 0.045 | 1.840 | 0.052 | 0.072 | 1.404 |
| hsa-miR-215-5p | 0.109 | 0.128 | 1.173 | 0.103 | 0.117 | 1.141 |
| hsa-miR-221-3p | 0.300 | 0.690 | 2.297 | 0.488 | 0.580 | 1.189 |
| hsa-miR-222-3p | 0.095 | 0.300 | 3.160 | 0.194 | 0.226 | 1.165 |
| hsa-miR-223-3p | 13.123 | 42.341 | 3.227 | 22.848 | 25.528 | 1.117 |
| hsa-miR-223-5p | 0.027 | 0.058 | 2.114 | 0.022 | 0.026 | 1.181 |
| hsa-miR-301a-3p | 0.030 | 0.087 | 2.908 | 0.059 | 0.064 | 1.094 |
| hsa-miR-320a | 0.792 | 1.499 | 1.892 | 1.252 | 1.448 | 1.157 |
| hsa-miR-320b | 0.375 | 0.709 | 1.892 | 0.588 | 0.739 | 1.257 |
| hsa-miR-320c | 0.440 | 0.873 | 1.986 | 0.739 | 0.949 | 1.283 |
| hsa-miR-326 | 0.037 | 0.044 | 1.181 | 0.147 | 0.090 | 0.611 |
| hsa-miR-328-3p | 0.071 | 0.108 | 1.505 | 0.119 | 0.153 | 1.292 |
| hsa-miR-331-3p | 0.057 | 0.074 | 1.292 | 0.066 | 0.084 | 1.283 |
| hsa-miR-335-3p | 0.011 | 0.024 | 2.144 | 0.012 | 0.012 | 1.014 |
| hsa-miR-335-5p | 0.014 | 0.025 | 1.753 | 0.006 | 0.018 | 3.160 |
| hsa-miR-338-3p | 0.052 | 0.093 | 1.778 | 0.095 | 0.090 | 0.953 |
| hsa-miR-339-3p | NA | 0.018 | NA | NA | 0.013 | NA |
| hsa-miR-339-5p | 0.029 | 0.050 | 1.717 | 0.226 | 0.089 | 0.392 |
| hsa-miR-342-3p | 0.370 | 0.635 | 1.717 | 0.388 | 0.622 | 1.602 |
| **hsa-miR-361-5p** | **0.049** | **0.272** | **5.540** | **0.085** | **0.153** | **1.803** |
| hsa-miR-362-3p | 0.004 | 0.013 | 3.317 | 0.013 | 0.021 | 1.636 |
| hsa-miR-363-3p | 0.117 | 0.244 | 2.085 | 0.189 | 0.197 | 1.042 |
| hsa-miR-365a-3p | 0.022 | 0.031 | 1.366 | 0.021 | 0.022 | 1.014 |
| hsa-miR-374a-5p | 0.232 | 0.385 | 1.659 | 0.284 | 0.261 | 0.920 |
| hsa-miR-374b-5p | 0.137 | 0.209 | 1.526 | 0.138 | 0.124 | 0.901 |
| **hsa-miR-375** | **0.074** | **0.036** | **0.493** | **0.083** | **0.041** | **0.497** |
| hsa-miR-376a-3p | 0.034 | 0.075 | 2.219 | 0.067 | 0.077 | 1.149 |
| hsa-miR-376c-3p | 0.030 | 0.064 | 2.099 | 0.052 | 0.074 | 1.404 |
| hsa-miR-382-5p | 0.024 | 0.042 | 1.765 | 0.017 | 0.037 | 2.158 |
| *Continued from page 2* | | | | | | |
| hsa-miR-409-3p | 0.023 | 0.048 | 2.071 | 0.025 | 0.017 | 0.683 |
| hsa-miR-421 | 0.011 | 0.013 | 1.181 | 0.011 | 0.021 | 1.879 |
| hsa-miR-423-3p | 0.120 | 0.335 | 2.789 | 0.635 | 0.388 | 0.611 |
| hsa-miR-424-5p | 0.292 | 0.215 | 0.737 | 0.212 | 0.147 | 0.693 |
| hsa-miR-425-3p | 0.028 | 0.085 | 3.053 | 0.080 | 0.060 | 0.747 |
| hsa-miR-454-3p | 0.024 | 0.049 | 2.042 | 0.037 | 0.066 | 1.765 |
| hsa-miR-483-5p | N.A. | 0.012 | N.A. | N.A. | N.A. | N.A. |
| hsa-miR-484 | 0.335 | 0.798 | 2.378 | 0.734 | 0.639 | 0.871 |
| hsa-miR-485-3p | 0.011 | 0.021 | 1.945 | 0.012 | 0.022 | 1.815 |
| hsa-miR-486-5p | 14.162 | 13.214 | 0.933 | 6.840 | 8.658 | 1.266 |
| hsa-miR-495-3p | 0.009 | N.A. | N.A. | 0.011 | N.A. | N.A. |
| hsa-miR-497-5p | 0.009 | 0.016 | 1.866 | 0.034 | 0.015 | 0.426 |
| hsa-miR-501-3p | 0.010 | 0.018 | 1.741 | 0.011 | 0.007 | 0.651 |
| hsa-miR-502-3p | 0.020 | 0.026 | 1.301 | 0.029 | 0.035 | 1.189 |
| hsa-miR-505-3p | 0.018 | 0.053 | 2.969 | 0.023 | 0.022 | 0.946 |
| hsa-miR-532-3p | 0.029 | 0.087 | 2.969 | 0.052 | 0.052 | 0.993 |
| hsa-miR-532-5p | 0.029 | 0.081 | 2.809 | 0.064 | 0.099 | 1.558 |
| hsa-miR-543 | N.A. | 0.010 | N.A. | N.A. | N.A. | N.A. |
| hsa-miR-574-3p | 0.021 | 0.027 | 1.292 | 0.021 | 0.042 | 1.986 |
| hsa-miR-584-5p | 0.028 | 0.051 | 1.778 | 0.034 | 0.050 | 1.474 |
| hsa-miR-590-5p | 0.035 | 0.050 | 1.434 | 0.026 | 0.061 | 2.362 |
| hsa-miR-629-5p | N.A. | 0.010 | N.A. | N.A. | 0.012 | N.A. |
| hsa-miR-652-3p | 0.128 | 0.311 | 2.428 | 0.167 | 0.265 | 1.591 |
| hsa-miR-660-5p | 0.041 | 0.114 | 2.751 | 0.054 | 0.058 | 1.064 |
| hsa-miR-766-3p | 0.036 | 0.043 | 1.197 | 0.075 | 0.061 | 0.807 |
| hsa-miR-874-3p | 0.013 | 0.022 | 1.717 | 0.034 | 0.040 | 1.157 |
| hsa-miR-877-5p | 0.008 | 0.009 | 1.028 | N.A. | 0.009 | N.A. |
| hsa-miR-885-5p | 0.013 | 0.026 | 1.972 | 0.051 | 0.035 | 0.683 |
| hsa-miR-1260a | 0.239 | 0.443 | 1.853 | 0.675 | 0.588 | 0.871 |
| hsa-miR-2110 | 0.011 | 0.026 | 2.250 | 0.017 | 0.022 | 1.310 |

**Table S5:** Analyzed parameters revealed significant changes for Resting Energy Expenditure (REE), FGF21, NEFA, Glc, Tsupra, and HR in individuals during acute cold exposure.
CE = cold exposure, Glc = fasting glucose, HR = heart rate, NEFA = Non-esterified Fatty Acids, REE = Resting Energy Expenditure, RQ = respiratory quotient, TN = thermoneutrality, TG = triglycerides, Tsupra = supraclavicular temperature, CRP = C-reactive protein,

|  |  | TN | | CE | |  |  |
| --- | --- | --- | --- | --- | --- | --- | --- |
| Parameter (Δ) | N | Mean | Std. Dev. | Mean | Std. Dev. | Regulation | Wilcoxon test p-value |
| REE (Kcal/d) | 169 | 1609 | 294.9 | 1726 | 360.4 | ↑ | < 0.0001 |
| RQ | 169 | 0.8 | 0.1 | 0.8 | 0.1 | ↓ | < 0.0001 |
| CRP (mg/l) | 168 | 0.2 | 0.4 | 0.3 | 0.4 | ↑ | < 0.0001 |
| HR (beats/min) | 169 | 68.6 | 11.8 | 62.6 | 10.3 | ↓ | < 0.0001 |
| Systolic blood preasure (mmHg) | 169 | 116.0 | 12.2 | 120.0 | 12.1 | ↑ | < 0.0001 |
| Diastolic blood preasure (mmHg) | 169 | 75.9 | 7.8 | 81.2 | 7.9 | ↑ | < 0.0001 |
| Glc (mg/dl) | 167 | 91.9 | 8.2 | 85.9 | 9.1 | ↓ | < 0.0001 |
| TG (mg/dl) | 168 | 82.9 | 39.9 | 92.0 | 41.1 | ↑ | < 0.0001 |
| fT3 (pmol/l) | 168 | 3.3 | 0.4 | 3.2 | 0.4 | ↓ | < 0.0001 |
| NEFA (µmol/l) | 168 | 292.3 | 127.6 | 521.8 | 219.3 | ↑ | < 0.0001 |
| Insulin (ng/ml) | 168 | 9.8 | 11.3 | 9.5 | 10.5 | ↓ |  |
| Adiponectin (µg/ml) | 168 | 7.7 | 5.2 | 8.7 | 6.8 | ↑ | < 0.0001 |
| Leptin (ng/ml) | 168 | 12.0 | 17.3 | 8.9 | 14.2 | ↓ | < 0.0001 |
| Tsupra (°C) | 168 | 35.7 | 0.6 | 35.7 | 0.8 | → |  |
| Tskin (°C) | 167 | 33.9 | 0.5 | 30.4 | 1.3 | ↓ | < 0.0001 |

**Table S6:** GTEx bulk tissue results of comparison VC and SC tissue

| Gene | median Sat n = 663 | median VAT n = 541 | median SAT log10(TPM+1) | median VAT log10(TPM+1) | **FOC** |
| --- | --- | --- | --- | --- | --- |
| ADIPOQ | 564.7 | 502.1 | 2.753 | 2.702 | **0.981** |
| ADRB3 | 0.06014 | 0.192 | 0.025 | 0.076 | **3.007** |
| CIDEA | 101.3 | 113.3 | 2.010 | 2.058 | **1.024** |
| DIO2 | 1.489 | 0.3758 | 0.396 | 0.139 | **0.350** |
| ELOVL3 | 2.016 | 2.37 | 0.479 | 0.528 | **1.101** |
| FABP4 | 6229 | 7084 | 3.794 | 3.850 | **1.015** |
| FGF21 | n.a. | 0.03368 | 0.000 | 0.014 | **n.a.** |
| LEP | 198.8 | 55.4 | 2.301 | 1.751 | **0.761** |
| PPARGC1a | 1.888 | 2.289 | 0.461 | 0.517 | **1.123** |
| PRDM16 | 2.385 | 1.48 | 0.530 | 0.394 | **0.745** |
| UCP1 | n.a. | 0.1676 | 0.000 | 0.067 | **n.a.** |

**Table S7:** Primer sequences used for qPCR

| Primer Name | Sequence (5´-3´orientation) |
| --- | --- |
| p53-for | CCTCAGCATCTTATCCGAGTGG |
| p53-rev | TGGATGGTGGTACAGTCAGAGC |
| Adiponectin-for | GGTGAGAAGGGTGAGAAAGGA |
| Adiponectin-for | ACACTGAATGCTGAGCGGTA |
| ADIPOR2-for | GCCTCTACATCACAGGAGCTGC |
| ADIPOR2-rev | CCTGGAGGTTTGAGACACCATG |
| ADRB3-for | AGCCCAGGCTTTGCCAACGGC |
| ADRB3-rev | GGGACTCATTCTGAACAGAGGC |
| CIDEA-for | TTGGGAGACAACACGCATT |
| CIDEA-rev | CGTTAAGGCAGCCGATGAAG |
| DIO2-for | TTGAGCCGCTCCAAGTCCACTC |
| DIO2-rev | CTGTACTGGAGACATGCACCAC |
| ELOVL3-for | CTACCTGGTTCTCATCGCTGTG |
| ELOVL3-rev | GTAGCACAGTCCCCATAATGCC |
| FABP4-for | CATGTGCAGAAATGGGATGGA |
| FABP4-rev | CGAACTTCAGTCCAGGTCAAC |
| FGF21-for  FGF21-rev | AGATGCGGTCGCTTCTTTCA  TCTGCGCCCCATCTGAATTT |
| GAPDH-for | GATCATCAGCAATGCCTCCTGC |
| GAPDH-rev | ACAGTCTTCTGGGTGGCAGTGA |
| IPO8-for | CGGATTATAGTCTCTGACCATGTC |
| IPO8-rev | TGTGTCACCATGTTCTTCAGG |
| Leptin-for | TTTGGCCCTATCTTTTCTATGTCC |
| Leptin-rev | TGGAGGAGACTGACTGCGTG |
| PPARGC1A-for | CCAAAGGATGCGCTCTCGTTCA |
| PPARGC1A-rev | CGGTGTCTGTAGTGGCTTGACT |
| PRDM16-for | TCCTGAAGACATTCCGATCC |
| PRDM16-rev | CCGAAGTCTGTCTCCTTTGC |
| UCP1-for | GGAGGCCTTTGTGAAAAACA |
| UCP1-rev | CTTGAAGAAAGCCGTTGGTC |

**Figure S1:** Relative expression levels of p53 and LDH measurements of transfected and cold exposed cells revealed no apoptosis of the cells.


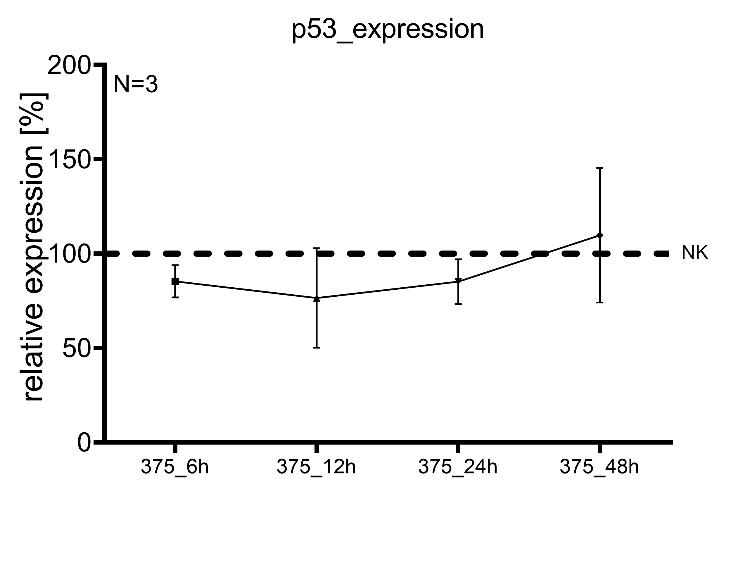


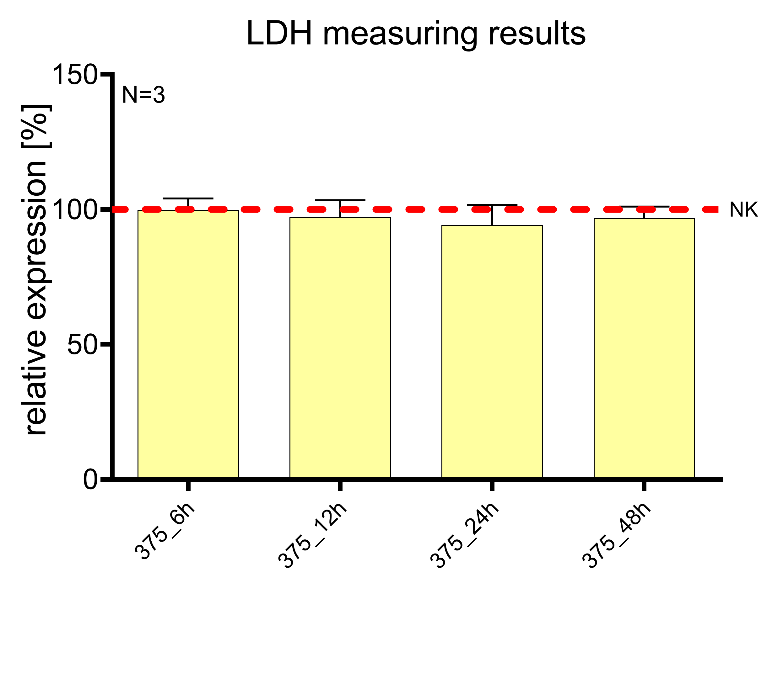

Supplement: Supplementary file 1 — Supplementary Information. [file 41598_2022_13610_MOESM1_ESM.docx]
